# NF-kappaB activation within macrophages leads to an anti-tumor phenotype in a mammary tumor lung metastasis model

**DOI:** 10.1186/bcr2935

**Published:** 2011-08-31

**Authors:** Linda Connelly, Whitney Barham, Halina M Onishko, Lianyi Chen, Taylor P Sherrill, Tahera Zabuawala, Michael C Ostrowski, Timothy S Blackwell, Fiona E Yull

**Affiliations:** 1 Department of Cancer Biology, Vanderbilt University, 771 Preston Research Building, 2220 Pierce Avenue, Nashville, TN 37232, USA; 2 Department of Pharmaceutical Sciences, UH Hilo College of Pharmacy, University of Hawaii at Hilo, 200 W. Kawili Street, Hilo, HI 96720, USA; 3 Division of Allergy, Pulmonary and Critical Care Medicine, Department of Medicine, Vanderbilt University, T-1217 Medical Center North, Nashville, TN 37232, USA; 4 Department of Molecular and Cellular Biochemistry, The Comprehensive Cancer Center, Ohio State University, 810 Biomedical Research Tower, 460 W. 12th Avenue, Columbus, OH 43210, USA; 5 Department of Veterans Affairs, T-1217 Medical Center North, Nashville, TN 37232, USA

## Abstract

**Introduction:**

Metastasis from primary tumor to the lungs is a major cause of the mortality associated with breast cancer. Both immune and inflammatory responses impact whether circulating mammary tumor cells successfully colonize the lungs leading to established metastases. Nuclear factor -kappaB (NF-κB) transcription factors regulate both immune and inflammatory responses mediated in part by the activities of macrophages. Therefore, NF-κB activity specifically within macrophages may be a critical determinant of whether circulating tumor cells successfully colonize the lungs.

**Methods:**

To investigate NF-κB signaling within macrophages during metastasis, we developed novel inducible transgenic models which target expression of the reverse tetracycline transactivator (rtTA) to macrophages using the *cfms *promoter in combination with inducible transgenics that express either an activator (cIKK2) or an inhibitor (IκBα-DN). Doxycyline treatment led to activation or inhibition of NF-κB within macrophages. We used a tail vein metastasis model with mammary tumor cell lines established from MMTV-Polyoma Middle T-Antigen-derived tumors to investigate the effects of modulating NF-κB in macrophages during different temporal windows of the metastatic process.

**Results:**

We found that activation of NF-κB in macrophages during seeding leads to a reduction in lung metastases. The mechanism involved expression of inflammatory cytokines and reactive oxygen species, leading to apoptosis of tumor cells and preventing seeding in the lung. Activation of NF-κB within macrophages after the seeding phase has no significant impact on establishment of metastases.

**Conclusions:**

Our results have identified a brief, defined window in which activation of NF-κB has significant anti-metastatic effects and inhibition of NF-κB results in a worse outcome.

## Introduction

The microenvironment that exists during chronic inflammation can contribute to cancer progression [1,2]. Therefore, anti-inflammatory strategies are being investigated as potential cancer therapies. However, such approaches may have undesired effects on host immune responses. Potentially, these negative effects could be limited by targeting interventions to specific cell types, but little is known about the contribution of individual inflammatory cell types to the progression of cancer.

The transcription factor nuclear factor kappa B (NF-κB) regulates inflammatory status and plays key roles in immune responses. NF-κB is a dimer formed from a multi-subunit family consisting of p65 (Rel A), Rel B, c-Rel, p105/p50 (NF-κB1), and p100/p52 (NF-κB2) [3,4]. In the classical NF-κB signaling pathway, the p50/p65 subunits are held in the cytosol by the inhibitory IκBα [5,6]. During activation, IκB kinase (IKK) 2 phosphorylates IκBα leading to ubiquitination and degradation. P50/p65 then translocates into the nucleus, binds to consensus DNA sequences activating expression of target genes [3,6,7]. We and others have modulated this signaling using a constitutive form of IKK2 to activate or a mutant form of IκBα to inhibit NF-κB activity [8-10].

Macrophages are key mediators of the interaction between inflammation, immunity and cancer. The role of macrophages in cancer has received attention due to the discovery of their tumor-promoting effects [11-13]. Efforts have been made to classify macrophages according to phenotype. The M1 macrophage phenotype is associated with production of reactive oxygen species (ROS), presentation of antigens, release of inflammatory cytokines and cytotoxic effects on pathogens and tumor cells. In contrast, the M2 macrophage phenotype has a reduced ability to present antigens and produce ROS, expresses scavenger receptors, promotes angiogenesis and wound healing, and is associated with tumor-promoting effects [14,15]. However, tumor-associated macrophages (TAMs) can express genes associated with both the M1 and M2 phenotypes highlighting the need for further investigation into macrophage phenotype during tumorigenesis [16].

Recent studies have investigated the role of NF-κB in macrophages during tumorigenesis. A LysM-Cre/floxed IKK2 transgenic, resulting in deletion of IKK2 in cells of the myeloid lineage (macrophages and granulocytes), led to a reduction in colon tumor incidence and size in a colitis-associated cancer model [17]. An IκB-super repressor targeted to Kupffer cells (liver macrophages) led to a similar reduction in tumor incidence in a murine model of hepatocellular carcinoma [18]. It has been reported that TAMs can induce invasive behavior of ovarian tumor cells via NF-κB [19,20]. In established orthotopic ovarian tumors, the introduction of macrophages with inhibited IKK2 led to a reduction in tumor burden. These effects were associated with a switch from M2 to M1 phenotype upon NF-κB inhibition [21]. These studies suggest a tumor-promoting effect of NF-κB in myeloid cells, including macrophages.

Despite these data pointing to a pro-tumor role for NF-κB in macrophages, genes regulated by NF-κB could also lead to an anti-tumor phenotype, suggesting that effects may be more complex. We have generated transgenic mouse models to modulate NF-κB in specific tissues by introducing doxycycline (dox) in drinking water [8,10]. To investigate NF-κB signaling within macrophages during metastasis we used a bi-transgenic system in which the colony stimulating factor 1 receptor promoter (cfms) drives the monocyte/macrophage-specific expression of the reverse tetracycline transactivator (rtTA). To activate NF-κB, the cfms-rtTA mouse was crossed with a second transgenic in which constitutively active IKK2 (cIKK2) was controlled by the tet operon (termed IKFM). To inhibit NF-κB, cfms-rtTA is crossed with a transgenic line in which dominant negative IκBα is controlled by the tet operon (termed DNFM). We used these models in a mouse mammary tumor cell tail vein-injection metastasis model that is extensively used as a methodology to investigate the later stages of the metastatic process from the point at which circulating tumor cells are present. We find that activation of NF-κB in macrophages during a short window around the time of cell injection leads to a reduction in lung metastasis of mammary tumor cells. The mechanism involves induction of an anti-tumor M1 phenotype that rapidly clears tumor cells. Two days after injection of tumor cells this window of opportunity closes and from this point activation of NF-κB no longer exhibits anti-tumor effects.

## Materials and methods

### Tumor cells

PyVT R221A and PYG 129 polyoma tumor cells were isolated from PyVT mammary gland tumors and cultured as previously described [9,22].

### Mouse strains

All animal experiments were approved by the Vanderbilt University Institutional Animal Care and Use Committee. All mice were on an FVB strain background, except the TG/CRE/FMR mice, which were mixed background (C57BL6 and FVB).

To generate the cfms-rtTA transgenic, the 7.2 kb mouse *cfms *promoter region was used to drive the expression of *rtTA-M2 *[23]. The *cfms-rtTA-M2 *transgenic construct was microinjected into mouse embryonic stem cells by standard methods. Progeny were screened for incorporation of transgene by southern blot and founder lines identified. Macrophage specific expression of rtTA was determined in mice transgenic for cfms-rtTA, tet-O-Cre and the ROSA26 LSL-lacZ allele [24]. cfms-rtTA mice were crossed with mice containing the NF-κB inhibiting (tet-O)_7_-IκBαDN-Myc-His construct or the NF-κB activating (tet-O) _7_-FLAG-cIKK2 construct [8,10]. Double transgenics were termed DNFM or IKFM, respectively. Littermates lacking one or both transgenes were used as controls.

To generate TG/Cre/FMR mice, Tomato Red/GFP reporter mice [25] were crossed with tet-O-Cre transgenic mice [26]. Double transgenic progeny were then crossed with cfms-rtTA mice to produce triple transgenic offspring containing all three transgenes. Littermates lacking the tet-O-Cre were used as controls.

### Tail vein metastasis model

To induce transgene expression mice were treated with 2 g/L dox (Sigma, St Louis, MO, USA) in drinking water. Sucrose (5%) was added to decrease the bitter taste of dox water. A red bottle was used to prevent light-induced degradation and water was replaced twice weekly. For metastasis studies, 1 × 10^6 ^polyoma tumor cells (PyVT R221A or PYG 129) in PBS were injected via the tail vein. Mice were treated with dox either one week prior to cells until sacrifice, one week prior to cells until two days post injection, or two days post injection of cells until time of sacrifice. Mice were sacrificed at two weeks (PyVT R221A) or five weeks (PYG129) post cell injection for surface lung tumor quantification. Mouse lungs were inflated with Bouin's fixative (RICCA Chemical, Arlington, TX, USA). After 24 hours of fixation, surface lung tumors were counted.

For seeding studies, mice were treated with dox for one week then 1 × 10^6 ^PyVT R221A tumor cells were injected via the tail vein. Mice were sacrificed one or six hours post cell injection and lungs harvested for real-time PCR and western blot analysis.

### RNA isolation and RT-PCR

Tissue was homogenized in Trizol (Invitrogen, Carlsbad, CA, USA) at sacrifice. For characterization, IKFM mice and controls were treated with dox for one week followed by intraperitoneal injection of 1.5 ml of 4% thioglycolate in PBS. At sacrifice, three days post injection, the peritoneal cavity was lavaged with PBS and cells pelleted.

RNA isolation and reverse transcription reactions were performed as described [10] for all studies. For studies utilizing real-time PCR analysis, an Applied Biosystems Stepone Plus Real-Time PCR system and SYBR Green PCR Master Mix (Applied Biosystems, Foster City, CA, USA) were used. Primers: polyoma middle T [27], TNF-α [10], CXCL9: FOR 5' GTGGTGAAATAAAAAGATCAGGGC 3', REV 5' AAGAGAGAAATGGGTTCCCTG 3'; CCL3: FOR 5' TGCCCTTGCTGTTCTTCTCT 3', REV 5' GATGAATTGGCGTGGAATCT 3'; mannose receptor: FOR 5' CAAGGAAGGTTGGCATTTGT 3', REV 5' CCTTTCAGTCCTTTGCAAGC 3'; arginase-1: FOR 5' ATGGAAGAGACCTTCAGCTAC 3', REV 5' GCTGTCTTCCCAAGAGTTGGG 3'; GAPDH: FOR 5' TGAGGACCAGGTTGTCTCCT 3', REV 5' CCCTGTTGCTGTAGCCGTAT 3'.

### Bone marrow derived macrophages

IKFM and DNFM mice were treated with dox (2 g/L) for one week and bone marrow-derived macrophages (BMDMs) isolated as described [28]. BMDMs were treated with dox (1 μg/ml) for 16 hours. RNA was isolated as above, and RT-PCR was completed with the following primers: IKK: FOR 5' GGAGCTCCACCGCGGTGCGG 3', REV 5' TCAGGGACATCTCGGGCAGC 3', and DN: FOR 5' CCTGGCTGTTGTCGAATACC-3', REV 5' GGTGATGGTGATGATGACCGG 3'. BMDMs were grown on microscope slides and treated with dox as above. Cells were fixed in 3% paraformaldehyde and stained for F4-80 (Invitrogen, Carlsbad, CA, USA), with DAPI (Sigma, St Louis, MO, USA).

### Western blotting

Whole cell lung extracts were prepared and western blot performed as previously described [10]. Antibody: Cleaved-Caspase-3 (Cell Signaling Technology, Beverly, MA, USA).

### Luminol imaging

Mice were treated with dox (2 g/L) for one week. L-012 (1.25 mg in 100 μl PBS, Wako Chemicals, Richmond, VA, USA) was intravenously injected into anesthetized mice. Mice were imaged with an intensified charge-coupled device camera (IVIS 200; Xenogen, Hopkinton, MA, USA). Light emission was detected as photon counts and analyzed by defining a standard area over the chest and determining total integrated photon intensity (Living Image software; Xenogen, Hopkinton, MA, USA).

### Flow cytometry

Mice were sacrificed, lungs perfused with cold PBS, minced and incubated in RPMI media containing 0.7 mg/ml collagenase XI (Sigma, St Louis, MO, USA) and 30 μg/ml DNAse I (Sigma, St Louis, MO, USA) for 40 minutes at 37°C. Digests were strained through a 70 micron filter. Cells were pelleted and treated with 1 ml red blood cell (RBC) lysis buffer (ACK buffer), washed, and re-suspended in PBS. Cells were blocked with anti-mouse CD16/CD32 antibody (eBioscience, San Diego, CA, USA) before staining with anti-mouse antibodies: CD45 (30-F11), Gr-1 (Ly-6G), CD11b (M1/70), CD11c (N418), and CD19 (all eBioscience, San Diego, CA, USA); F4/80 and B220 (Invitrogen, Carlsbad, CA, USA); CD4 and NK1.1 (BD Pharmingen, San Diego, CA, USA). Analysis was performed on an LSRII cytometer with DIVA software (BD Biosciences, Franklin Lakes, NJ, USA).

### CD11b positive cell isolation

Mice were treated with dox (2 g/L) for one week prior to sacrifice. Lungs were digested as described above. Cell suspensions were incubated with MACS CDllb MicroBeads (Miltenyi Biotec, Auburn, CA, USA) and separated by positive selection using MS columns.

### Immunofluorescence

For whole lung tissue staining of IKFM and control mice, lungs were first perfused with PBS, then inflated and fixed in formalin overnight followed by paraffin embedding and sectioning. For cytospin staining, lungs were dissociated and CD11b+ cells positively selected as above. Cells were spun onto microscope slides then fixed in 3% paraformaldehyde. Antigen was unmasked by the sodium citrate method. Primary antibody: NF-κB phospho-p65 (Ser536) (Cell Signaling Technology, Beverly, MA, USA). Secondary antibody: goat anti-rabbit Alexa Fluor 594 (Invitrogen, Carlsbad, CA, USA). DAPI (Sigma, St Louis, MO, USA) was used for nuclei staining. Staining was visualized by Zeiss microscope and analyzed by MetaMorph software (Molecular Devices, Sunnyvale, CA).

TG/Cre/FMR lungs were perfused with cold PBS and then inflated and fixed for four to six hours in 4% paraformaldehyde before being paraffin embedded and sectioned. DAPI (Sigma, St Louis, MO, USA) counterstain was used as above.

### Statistical analyses

Statistical analyses were performed using Graph Pad Prism (GraphPad Software Inc., La Jolla, CA, USA). All data are plotted graphically with vertical bars representing standard error. Unpaired Student's *t *test was used to assess differences between experimental conditions. A probability (*P*) value of less than 0.05 was taken as an appropriate level of significance.

## Results

### Characterization of a novel transgenic model to activate or inhibit NF-κB activity in macrophages

To activate or inhibit NF-κB signaling in macrophages we used pairs of transgenics and the tet-on system. The first transgenic contains the colony stimulating factor receptor 1 promoter-reverse tetracycline transactivator (cfms-rtTA) leading to rtTA protein expression in macrophages. To generate IKFM mice, the cfms-rtTA is crossed with a transgenic containing the tet operon promoter element upstream of a FLAG-tagged cIKK2 gene. For DNFM mice, the cfms-rtTA is crossed with a transgenic containing the tet operon promoter element upstream of a myc-tagged dominant negative IκBα (DN-IκBα) gene [8-10]. Treatment of IKFM or DNFM with dox results in expression of transgene in macrophages. Littermates lacking one or both transgenes were used as controls.

**Figure 1:**
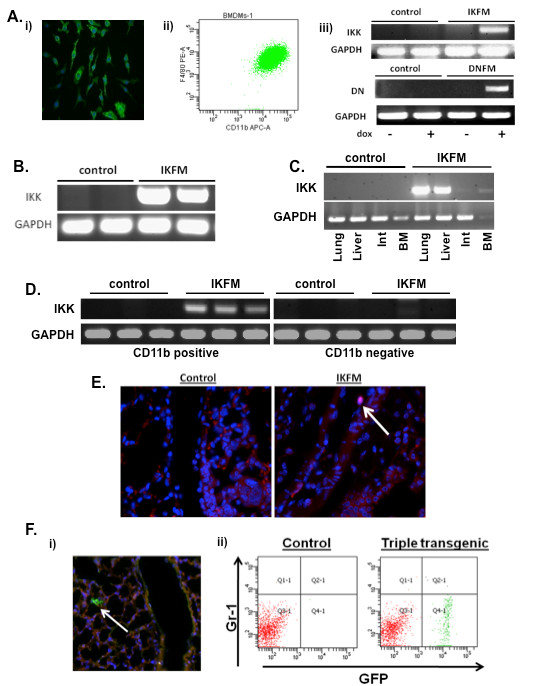
**Characterization of IKFM and DNFM mouse models**. **(a) **Bone marrow-derived macrophages were isolated from IKFM and DNFM mice and (i) stained for F4/80 via immunofluorescent staining (green = F4/80 and blue = DAPI), (ii) subjected to flow cytometric analysis of F4/80 and CD11b and (iii) treated with 1 μg/ml doxycycline (dox) for 24 hours *in vitro *and gene expression analyzed via RT-PCR. **(b) **Peritoneal macrophages were isolated from IKFM mice treated with dox (2 g/L) for one week and transgene expression analyzed via RT-PCR. **(c) **IKFM mice and controls were treated with dox (2 g/L) for four weeks and transgene expression was detected by RT-PCR in lung, liver, intestines (Int), and bone marrow (BM). **(d) **IKFM and control mice were treated with dox (2 g/L) for one week and CD11b positive and negative populations isolated from lungs for RT-PCR analysis of IKK. GAPDH was performed as control. **(e) **Phospho-p65 (Ser536) immunofluorescent staining (red = phospho-p65 and blue = DAPI) of lungs from IKFM and control mice bearing lung metastases and treated with dox (2 g/L). **(f) **(i) Lung sections of TG/Cre/FMR mice treated with dox for one week (blue = DAPI) and (ii) flow analysis of TG/Cre/FMR and control lungs. Total cells were first sorted for CD45 and CD11b double positive cells. This double positive population was then further sorted for Gr-1 and GFP, as shown.

BMDMs were cultured from IKFM or DNFM mice and controls. Immunohistochemistry for the macrophage-specific marker F4/80 (Figure 1a, part i) and flow cytometry for both F4/80 and CD11b (Figure 1a, part ii) confirmed that the cells were macrophages. Cells were treated with dox (1 μg/ml) for 24 hours, RNA isolated and RT-PCR performed for the expression of cIKK2 mRNA or DN-IκBα mRNA. cIKK2 expression was observed in IKFM macrophages and DN-IκBα was observed in DNFM macrophages, with no transgene expression observed in controls (Figure 1a, part iii). Primers were designed such that the reverse primer was in the FLAG sequence (cIKK2) or myc sequence (DN-IκBα), ensuring specificity for transgene expression.

To test *in vivo *expression of transgenes in macrophages, IKFM or control mice were treated with dox (2 g/L) for one week. Thioglycolate-elicited peritoneal macrophages were isolated and RT-PCR performed for cIKK2 transgene expression. Transgene mRNA was expressed in IKFM macrophages but not in controls (Figure 1b). To further characterize transgene expression *in vivo*, IKFM or control mice were treated with dox (2 g/L) for four weeks. Tissues with high levels of macrophages were isolated and RT-PCR performed for expression of the cIKK2 transgene (Figure 1c). Transgene expression was observed in tissues from IKFM mice and was absent in controls. Equivalent characterization was completed in DNFM peritoneal macrophages and tissues that also demonstrated macrophage-targeted, inducible DN transgene expression.

To further determine both the macrophage cell specificity and the activity associated with the transgene expression, we utilized several methods. First, lungs from IKFM mice or controls treated with dox for one week were homogenized and CD11b-positive and negative cells were separated. Using RT-PCR, cIKK2 expression was observed in CD11b-positive cells from the lungs of IKFM mice but not in CD11b-positive cells from control mice or in CD11b-negative cells from IKFM mice (Figure 1d). Furthermore, equal numbers of CD11b-positive cell populations from IKFM or control lungs were spun onto microscope slides and then stained and analyzed for nuclear phospho-p65 (Ser536). The CD11b-positive cells isolated from IKFM lungs showed an almost eight fold increase in the number of phospho-p65-positive macrophages vs. the number of positive macrophages in control CD11b-positive cells (7.74 ± 0.95 fold increase, *n *= 4 control and *n *= 4 IKFM, *P *= 0.0028), indicating that cIKK2 transgene expression is indeed increasing NF-κB signaling.

To confirm the effect of the transgene on NF-κB signaling *in vivo*, IKFM mice and controls were tail vein injected with PyVT R221A polyoma tumor cells. Mice were treated with dox beginning two days post injection until the time of sacrifice two weeks later, at which time all mice had similar lung tumor burden. Paraffin-embedded tissue was sectioned and stained for phospho-p65 (Ser536) expression as an indicator of NF-κB activation. A subset of macrophages within the IKFM lungs stained positive for nuclear phospho-p65, whereas none of these brightly stained macrophages were observed in control mice (Figure 1e). As an alternative approach to further confirm the cell specificity of the cfms-rtTA expression, we utilized a Tomato-Red/green fluorescent protein (GFP) reporter mouse crossed with a tet-O-Cre transgenic and the cfms-rtTA mouse. When treated with dox (2 g/L), triple transgenic mice, termed TG/Cre/FMR, reveal which cells express the cfms-rtTA, as the rtTA/Cre combination within these cells flips the red fluorescence to green GFP. Sections of lung tissue from TG/Cre/FMR mice on dox for one week confirm that cells identified by morphology as macrophages are flipped to green (Figure 1f; no GFP-positive cells were observed in control littermates that lacked the tet-O-Cre). Flow cytometry of lungs from TG/Cre/FMR mice and controls verifies that the GFP-positive cells are CD45^+^CD11b^+ ^and Gr1^- ^(Figure 1f). GFP expression was undetectable in CD45^+ ^Gr1^+ ^CD11b^- ^cells (data not shown).

### Activation of NF-κB activity in macrophages leads to a reduction in tumor burden in a tail vein metastasis model

**Figure 2:**
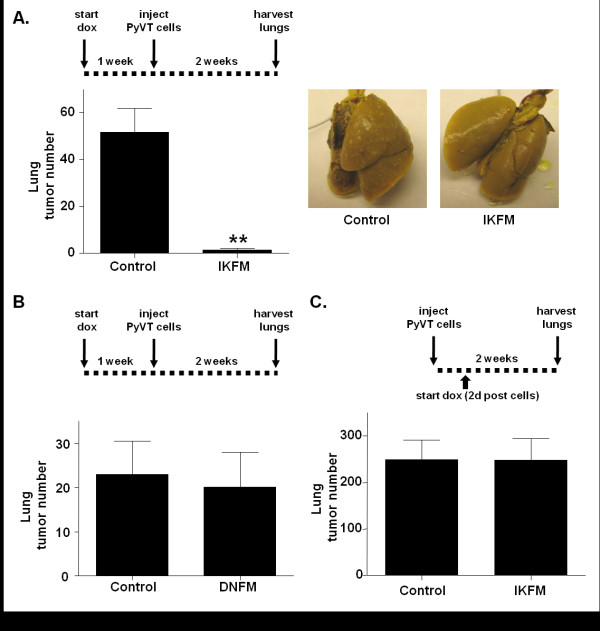
**Activation of NF-κB in macrophages results in fewer lung metastases**. IKFM or DNFM mice and controls were treated with doxycycline (dox; 2 g/L) for one week prior to PyVT cell injection. Dox treatment was continued until sacrifice at two weeks post-injection. **(a) **Lung tumor counts in IKFM and control mice (*P *= 0.0005; *n *= 11 control, *n *= 8 IKFM) and representative lung images. **(b) **Lung tumor counts in DNFM and control mice (*P *= 0.7997; *n *= 6 control, *n *= 8 DNFM). **(c) **IKFM mice were injected with PyVT cells. Dox treatment (2 g/L) began two days post injection and continued until sacrifice at two weeks post cells when surface lung tumors were counted (*P *= 0.9880; *n *= 7 control, *n *= 6 IKFM).

IKFM mice or controls were treated with dox for one week before injection of PyVT R221A cells. Dox treatment was continued until lungs were harvested two weeks post-cell injection. Analysis of surface lung tumor numbers revealed a significant reduction in lung tumors in IKFM mice as compared to controls (Figure 2a). The same experiment was performed in DNFM mice and controls, but no significant difference in lung tumor number was observed when NF-κB was inhibited (Figure 2b).

To investigate modulation of NF-κB during later stages of metastasis, PyVT R221A cells were injected in the tail vein of IKFM mice and controls and dox treatment was started two days after injection and continued until collection at two weeks post-injection. No significant difference was observed in tumor number between IKFM mice and controls (Figure 2c) suggesting that the anti-tumor effect may be an earlier event. Additionally, DNFM mice were injected with PyVT R221A cells, and dox treatment was started two days after injection and continued until sacrifice. Upon collection, DNFM and control mice showed no significant difference in lung tumor number (control 67.00 ± 27.08, *n *= 7; DNFM 45.44 ± 16.97, *n *= 9, *P *= 0.4924;). Thus, in this model there is no evidence of a pro-metastatic phenotype mediated via NF-κB activity in macrophages from either IKFM or DNFM studies in which modulation occurs from two days post cell injection.

### Reduction of lung metastasis by NF-κB in macrophages is an early event

**Figure 3:**
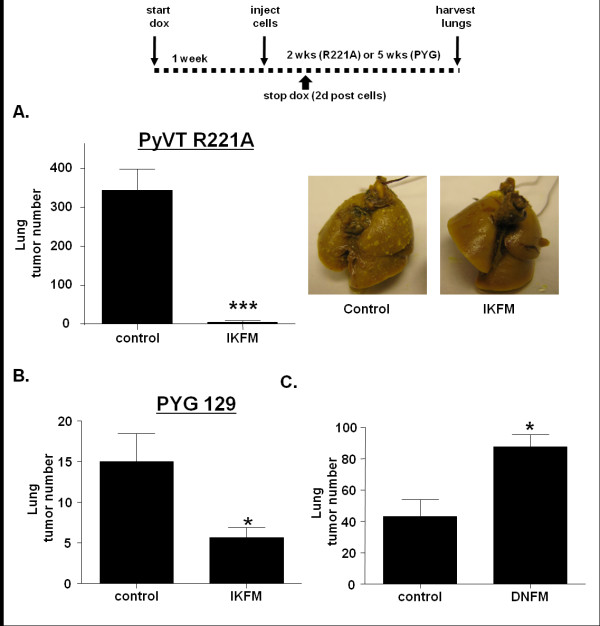
**Reduction of lung metastasis by NF-κB in macrophages is an early event**. IKFM or DNFM mice and controls were treated with doxycycline (dox; 2 g/L) for one week prior to cell injection. Dox treatment ended two days post cell injection and mice were sacrificed at two weeks post cells (PyVT R221A) or five weeks post cells (PYG 129). **(a) **Lung tumor counts in IKFM injected with PyVT R221A cells (*P *< 0.0001; *n *= 10 control, *n *= 7 IKFM) and representative lung images. **(b) **Lung tumor counts in IKFM injected with PYG 129 cells (*P *= 0.0270; *n *= 9 control, *n *= 8 IKFM). **(c) **Lung tumor counts in DNFM injected with PyVT R221A cells (*P *= 0.0039; *n *= 6 control, *n *= 9 DNFM).

As no effect was observed when dox treatment was started two days after cell injection, experiments were performed to look at the impact of NF-κB activation in macrophages in the period immediately surrounding tumor injection. IKFM mice and controls were treated with dox for one week before injection of PyVT R221A cells. Dox treatment was stopped two days post-injection and lungs were harvested two weeks post-injection. A significant reduction was observed in the number of lung tumors in IKFM compared with controls (Figure 3a). To ensure that this was not a cell line-specific effect, this experiment was repeated with a second, separately-derived mammary tumor cell line, PYG 129, that forms lung tumors five weeks after tail vein injection. IKFM mice and controls were treated with dox for one week before injection of PYG 129 cells via the tail vein. Dox treatment was stopped two days post-injection and lungs were harvested and surface tumors counted five weeks post-injection. A significant reduction in lung tumor formation was observed in IKFM mice as compared with control mice (Figure 3b). This indicates that the anti-tumor impact of NF-κB activation in macrophages occurs in the early stages of lung metastasis formation.

This experiment was repeated in DNFM mice to determine impact of NF-κB inhibition in macrophages on the early stages of lung metastasis formation. DNFM mice and controls were treated with dox for one week before injection of PyVT R221A cells. Dox treatment was stopped two days post-cell injection and lungs were harvested and surface tumors counted two weeks post-injection. A significant increase in tumor burden was observed in DNFM mice compared with controls (Figure 3c) confirming that NF-κB activity in macrophages blocks lung metastasis formation.

### Activation of NF-κB in macrophages leads to a shift in macrophage populations in the lung

**Figure 4:**
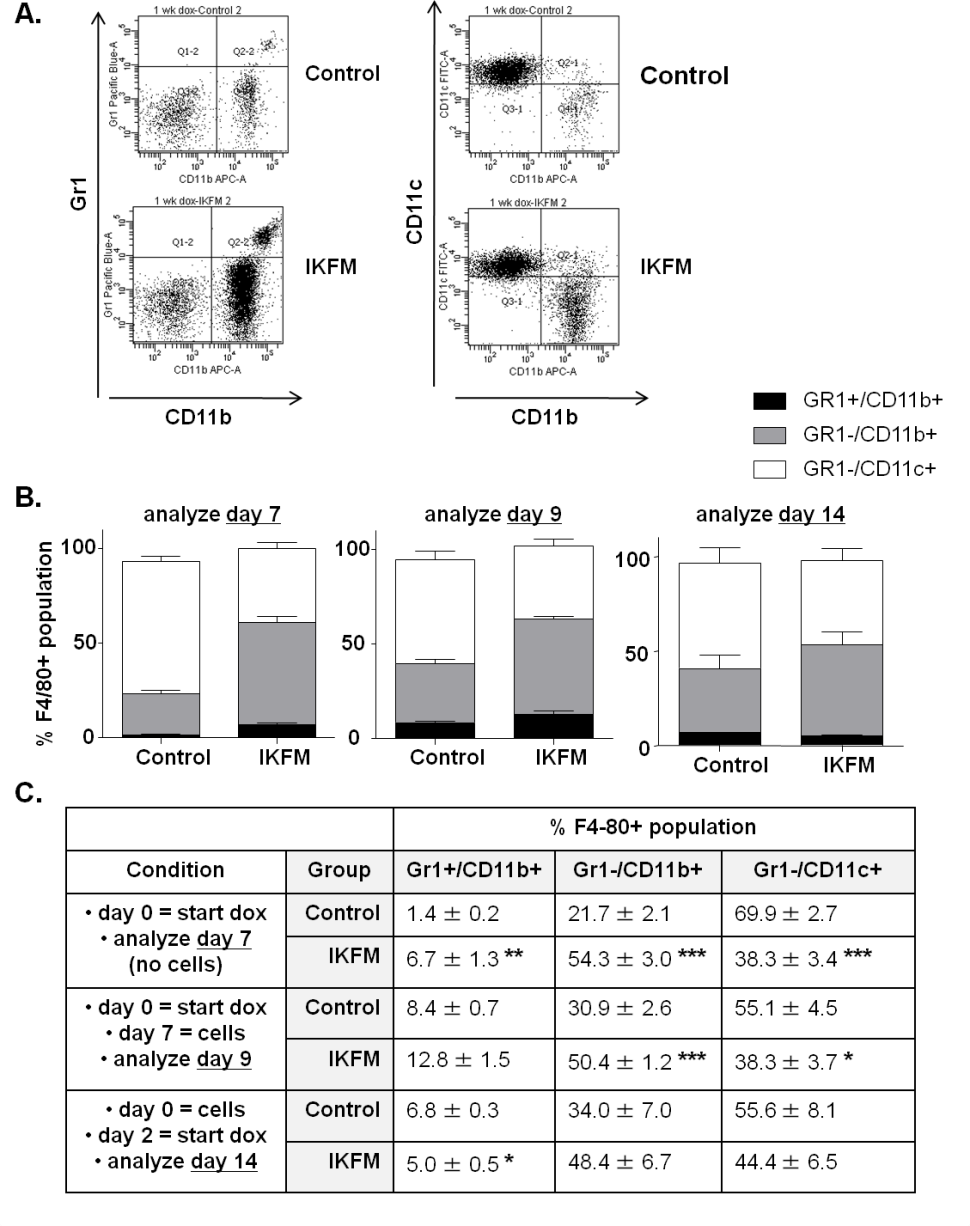
**Activation of NF-κB in macrophages leads to a shift in macrophage populations in the lung**. Flow cytometric analysis of dissociated lung cells from IKFM and control mice. Three treatment groups (all 2 g/L doxycycline (dox)): one week dox - mice were on dox for one week (control *n *= 4, IKFM *n *= 4); two days dox - mice were on dox for one week, PyVT R221A cells were injected via tail vein, dox treatment was stopped two days after injection and mice sacrificed (control *n *= 3, IKFM *n *= 4); two weeks dox - mice were injected with PyVT cells via tail vein, two days later mice were put on dox and two weeks after cell injection mice were sacrificed (control *n *= 3, IKFM *n *= 3). All cells were gated for CD45+ and then for F4/80+ cells. **(a) **Representative flow diagrams of one week dox treatment group. **(b) **Graphs and **(c) **table showing percentage of cell populations that were Gr1+/CD11b+ (black), Gr1-/CD11b+ (grey) and Gr1-/Cd11c+ (white; *P < 0.05, **P < 0.01, ***P < 0.001; n ≥ 3 for all groups).

To assess the environment that the tumor cells encounter upon being injected into the IKFM mice, flow cytometric analysis was performed to look at macrophage populations in IKFM lungs compared with controls. Mice were treated with dox for one week, lungs were homogenized and CD45+ (leukocyte marker), F4/80+ (macrophage marker) cells were selected for further cell surface marker analysis. Three different cell populations were analyzed within this group: Gr1+/CD11b+ reported to represent immature myeloid cells including myeloid-derived suppressor cells [29,30], Gr1-/CD11b+ reported to represent newly recruited/mature macrophages [31] and Gr1-/CD11c+, which are reported to represent lung resident macrophages [32]. Although total F4/80 positive populations were not significantly different (Control 25.5 ± 13.3, IKFM 30.9 ± 2.8 *n *= 4, *P *= 0.45, percentages expressed out of total CD45^+ ^cells), a significant increase in the percentage of Gr1+/CD11b+ as well as Gr1-/CD11b+ cells was observed in IKFM as compared with control lungs. A significant decrease in Gr1-/CD11c+ cells was also observed in IKFM lungs (Figure 4). Thus, activation of NF-κB in macrophages correlates with a shift in the macrophage population surface markers in the lung environment that suggests a population consisting of immature and recruited/mature cells with a lower proportion of resident lung macrophages even in the absence of tumor cells. A similar change in cell populations was observed in IKFM mice treated with dox for one week followed by cell injection and harvest of lungs two days post-injection. Therefore, this phenotype correlates with both time points at which a significant reduction in surface lung tumor formation was observed. A somewhat similar shift in monocyte populations is observed in control mice, but only after cell injection, whereas the changes occur in IKFM lungs with dox treatment alone. This suggests that activating NF-κB in macrophages "pre-educates" the IKFM lung environment to become anti-tumor, an effect that is achieved in control mice to a lesser degree only in response to tumor cells. These differences in macrophage populations were not observed in IKFM mice compared with controls when cells were injected, dox treatment started two days post-injection and lungs harvested for analysis two weeks post-injection, correlating with the lack of effect on lung tumor number. Analysis of markers of other immune cell types within the lungs after one week of dox treatment indicates that the percentages of CD4^+ ^T cells and B220^+^/CD19^+ ^B cells are decreased in IKFM whereas the NK1.1^+ ^natural killer cell population is increased (CD4^+ ^T cells: control = 39.3 ± 2.6 and IKFM = 22.4 ± 0.7, *P *= 0.0001; B220^+^/CD19^+ ^B cells: control = 15.1 ± 1.5 and IKFM = 10.1 ± 1.0, *P *= 0.0123; NK1.1^+ ^natural killer cells: control = 2.9 ± 0.4 and IKFM = 5.6 ± 0.5, *P *= 0.0024; *n *= 6 control, *n *= 8 IKFM for all markers and values expressed as percentage of CD45^+ ^cells). These changes are again suggestive of a pre-activated innate immune response that occurs prior to tumor cell injection.

### Expression pattern in IKFM lung homogenates suggests that the majority of macrophages are M1 phenotype

**Figure 5:**
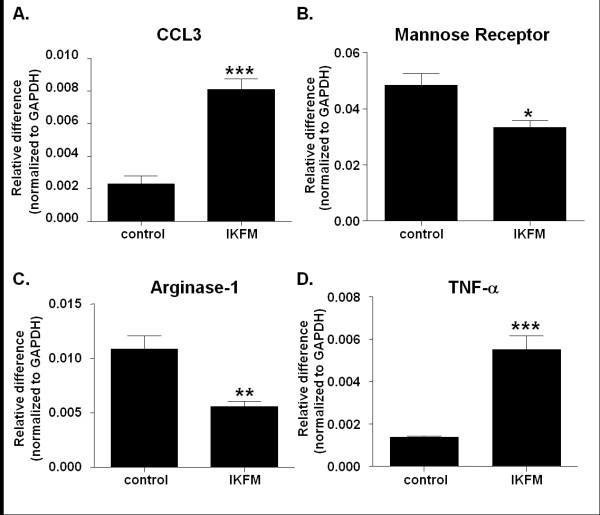
**Expression pattern in IKFM lung homogenates suggests that the majority of macrophages are M1 phenotype**. RNA was isolated from whole lung samples of IKFM and control mice treated with doxycycline (dox; 2 g/L) for one week. Quantitative real-time PCR was performed for **(a) **CCL3, **(b) **mannose receptor, **(c) **arginase-1 and **(d) **TNF-alpha (*P *< 0.05, **P < 0.01, ***P < 0.001; *n *= 5 control, *n *= 5 IKFM).

To determine whether this reduction in lung metastases and alteration in macrophage populations correlated with a particular macrophage phenotype, quantitative real-time PCR was performed for M1 and M2 markers. IKFM and controls were treated with dox for one week after which total lung RNA was extracted and real-time PCR performed. An increase was observed in CCL3 and TNF-α mRNA while a decrease was observed in arginase-1 and mannose receptor mRNA (Figure 5). The decrease in mannose receptor expression was confirmed in CD11b-positive cells isolated from IKFM vs. control lungs, assessed via real-time PCR (*n *= 4 control, *n *= 4 IKFM, *P *= 0.003). Taken together, this suggests that in the lungs of IKFM mice M1 represents the dominant macrophage phenotype [33].

### A reduction in tumor cell seeding accompanied by an increase in apoptotic signaling is observed in lungs from IKFM mice

**Figure 6:**
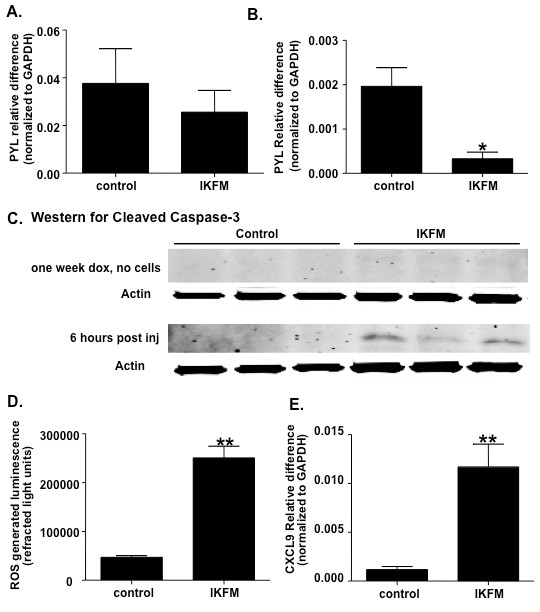
**A reduction in tumor cell seeding is observed in lungs from IKFM mice**. IKFM and control mice were treated with doxycycline (dox; 2 g/L) for one week before injection of PyVT R221A cells. Lungs were harvested at **(a) **one hour (*n *= 5 control, *n *= 7 IKFM, *P *= 0.4410) and **(b) **six hours (*n *= 3 control, *n *= 3 IKFM, *P *= 0.0214) post injection and RNA isolated from whole lung samples for quantitative real-time PCR analysis of Polyoma (PYL) expression. **(b) **Western blot for cleaved caspase-3 on whole cell lung homogenates from mice treated with dox for one week (no cells injected; *n *= 4 control and *n *= 4 IKFM) and homogenates from the six hour time point (*n *= 4 control and *n *= 4 IKFM). **(c) **IKFM mice and controls were treated with dox for one week and then imaged for ROS (*n *= 3 control and *n *= 3 IKFM; *P *= 0.0011). **(e) **Real-time PCR analysis of whole lung RNA for CXCL9 (*n *= 4 control, *n *= 3 IKFM, *P *= 0.0033).

The effects on seeding of tumor cells after intravenous injection were investigated. IKFM and controls were treated with dox for one week before injection of PyVT R221A cells. Lungs were harvested at one hour and six hours post-injection and RNA isolated. Quantitative real-time PCR was performed for the polyoma transgene, which reflects tumor cell levels in the lungs. At one hour post-injection there was no significant difference in levels of polyoma mRNA between control and IKFM (Figure 6a). At six hours post-injection a significant reduction in polyoma mRNA was observed in IKFM lungs compared with control (Figure 6b). This suggests that there were less PyVT R221A cells in IKFM lungs than control at this point, perhaps due to an increased level of clearance, which would correlate with a lack of tumor formation in these mice.

To investigate whether the environment in IKFM lungs increased apoptosis of PyVT R221A cells, western blot for activated caspase 3 was performed in whole lung homogenates. Caspase 3 activation was undetectable in the lungs of IKFM mice treated with dox for one week. However, a higher level of caspase 3 activation was observed in lungs from IKFM mice (Figure 6c) at six hours post cell injection indicating increased levels of apoptosis. This suggests that activation of NF-κB does not result in significant cell death in the absence of injected tumor cells and implies that the apoptosis occurs in the tumor cells as opposed to the lung epithelium.

### Increased clearing of tumor cells and apoptosis in IKFM lungs correlates with higher ROS levels and increased CXCL9 expression

As an increased clearance of tumor cells had been observed, we were interested in the mechanism contributing to cytotoxic effects. Therefore, we investigated whether there was a change in levels of ROS. IKFM mice and controls were treated with dox for one week and then injected with the L-012 luminol derivative to allow *in vivo *imaging of ROS levels over the chest area. IKFM mice showed a significant increase in ROS levels as compared with controls (Figure 6d). This suggests that the lung environment in IKFM mice was more cytotoxic to tumor cells correlating with increased clearance and a reduction in final tumor counts.

To further investigate the lung microenvironment at the time of tumor injection, cytokine profiling of bronchial alveolar lavage (BAL) fluid from IKFM mice and controls after one week of dox treatment was performed using the R&D Systems Proteome Profiler mouse cytokine panel array kit. The only significant difference detected was an increase in levels of CXCL9 (monokine induced by gamma interferon) in the IKFM fluid (data not shown). To confirm this change, real-time PCR was performed on lung mRNA from the same time point. There was a significant increase in expression levels of CXCL9 (Figure 6e), which correlates with an anti-tumor environment [34].

## Discussion

We generated a transgenic mouse model to investigate the role of classical NF-κB signaling in macrophages during tumorigenesis. We show that activation of NF-κB in macrophages in a mammary tumor tail vein metastasis model leads to a reduction in lung tumor formation with effects observed only when NF-κB is modulated prior to tumor cell introduction during the early seeding phase. Investigations into the lung phenotype associated with this effect show that activation of NF-κB leads to a shift in macrophage populations in the lung with a higher percentage of cells that are Gr1+/Cd11b+ and Gr1-/CD11b+ and a lower percentage of Gr1-/Cd11c+ cells. This shift in macrophage population and reduction in lung tumor numbers occurs in lungs that express increased levels of markers of the M1 anti-tumor macrophage phenotype. In agreement with this, we see a decrease in mammary tumor cell seeding following cell injection, an increase in apoptosis and enhanced formation of ROS and the cytokine CXCL9. Our data suggest that activation of NF-κB in macrophages within the lung during the seeding of lung metastases has an anti-tumor effect.

Our data are in contrast with other *in vivo *studies highlighting a tumor-promoting role for macrophages [17,21]. This may be due to the different murine models and cancer types and the timing of NF-κB activation. These recently published studies have used the strategy of Cre-mediated deletion of the IKK2 to inhibit NF-κB signaling in myeloid cells. Our strategy employs specific activation of the signaling pathway by expression of a constitutive activator or direct inhibition by expression of a dominant form of inhibitor, either of which is regulated in an inducible manner. In our model, activation of NF-κB in macrophages before tumor cell arrival generates a hostile environment for tumor seeding and growth. This environment is similar to the M1-type macrophage response seen with a bacterial challenge [33]. The tail vein metastasis model is inherently an acute model as mice develop a heavy metastatic load in the lungs at two weeks and need to be euthanized for humane reasons. This likely precludes observing the pro-tumor effects that have been reported in models of established tumors. Future studies to address effects on established tumors will need to be performed using orthotopic models or those in which spontaneous tumors arise, such as the polyoma transgenic model, to address this issue.

Recent studies highlight the complexity of NF-κB signaling during tumorigenesis. Tumor-associated macrophages isolated from an orthotopic fibrosarcoma model show a defective activation of NF-κB in response to lipopolysaccharide [16]. Further investigation reveals that this lack of activation is due to p50 subunit homodimers and knockout of p50 restores the M1 macrophage phenotype and reduces tumor burden [35]. Although this study suggests that defective NF-κB activation within macrophages leads to the development of an M2, pro-tumor phenotype, others find that NF-kappaB signaling, specifically IKK2, is necessary to maintain the M2 phenotype in an ovarian cancer model [21]. These apparent contradictions were recently reviewed by Hagemann *et. al *[36] who propose that NF-κB regulation of TAMs is both context- and gene-dependent.

IKFM mice showed a significant shift in macrophage populations when NF-κB was activated for one week before tumors were established. Interestingly, when cells were analyzed at the third time point (where metastases are evident), a different pattern of surface marker expression was observed. This pattern was more similar to control populations. This agrees with recent reports showing the plasticity of the macrophage population during tumorigenesis with a different set of markers expressed at each stage [37].

The cell marker phenotype in the IKFM mice when an anti-tumor effect was observed correlates with the reduction in tumor burden. For example, GR1+/CD11b+ myeloid cells are a source of ROS, the levels of which were seen to increase [29]. CXCL9 was significantly up-regulated in IKFM lungs after one week of dox treatment. NF-κB activation leading to CXCL9 production within macrophages has previously been reported [38]. The injection of a mammary tumor cell line stably expressing CXCL9 cDNA into mice resulted in smaller tumors as well as fewer lung metastases. This was found to be a T-cell-mediated effect, in that the CXCL9 overexpressing tumors had increased levels of infiltrating CD4 T cells as compared with vector controls [34]. Although we see increased CXCL9 expression in our model, we observed a concurrent decrease in CD4 T cells as assessed by flow cytometry. This could imply a separate, non-T-cell-mediated, anti-tumor effect associated with CXCL9, or suggest that the time points analyzed were not optimal for analysis of lymphocyte infiltration. In either case, further investigation into the role of CXCL9 in this model is required.

## Conclusions

We have generated a novel pair of transgenic models that enable NF-κB to be either activated or inhibited in an inducible manner in macrophages/monocytes by administration of dox in the drinking water. These models will enable investigations of the role of NF-κB within macrophages in defined temporal windows within tumor development and progression and will potentially be of use for investigations of other disease processes, such as atherosclerosis. In the current study the models have been used to investigate the effects of modulation of NF-κB during the seeding phase of metastasis. In contrast to what may have been predicted from the currently available literature, our data show that activation of NF-κB in a short, defined window inhibits metastasis. In addition, inhibition of NF-κB may actually contribute to increased metastasis. Given the recent interest in developing NF-κB inhibitors for clinical cancer therapy [39] it is intriguing that, in our study, inhibition of NF-κB within macrophages during metastatic seeding resulted in a significant increase in lung tumor burden. This data suggest that inhibition in macrophages during tumorigenesis at certain time points may interfere with host-suppressive effects on metastasis formation, and demonstrates that timing and cell specificity may be the key determinants of the impact of NF-κB inhibitors as a cancer therapy.

## Abbreviations

BAL: bronchoalveolar lavage; BMDMs: bone marrow-derived macrophages; CCL3: chemokine (C-C motif) ligand 3; CD11b: cluster of differentiation molecule 11B; cfms: colony stimulating factor -1 receptor promoter; cIKK2: constitutive form of inhibitor of nuclear factor kappa-B kinase subunit beta; CXCL9: chemokine (C-X-C motif) ligand 9; DNFM: double transgenic mice which express dominant negative IKBα in macrophages in the presence of doxycycline; dox: doxycycline; ICCD: intensified charge coupled device; IκBα-DN: inhibitor of nuclear factor kappa B alpha dominant negative; IKFM: double transgenic mice which express cIKK2 in macrophages in the presence of doxycycline; LysM-cre: mouse strain expressing Cre recombinase from the endogenous *Lyzs *locus; NF-κB: nuclear factor kappa B; PBS: phosphate-buffered saline; PyVT: polyoma middle T antigen mouse model; ROS: reactive oxygen species; RT-PCR: reverse transcriptase polymerase chain reaction; rtTA: reverse tetracycline transactivator; TAM: tumor-associated macrophages; TNF-alpha: tumor necrosis factor alpha.

## Competing interests

The authors declare that they have no competing interests.

## Authors' contributions

LC performed initial characterization of the model, completed various trials of the tail vein studies, and drafted the manuscript. WB participated in ongoing breeding of the transgenic mice, completed tail vein trials and carried out real-time PCR, western-blot analysis and CDllb bead separation experiments. HO completed immunoflourescent staining, flow-cytometry experiments and analysis, CDllb bead separation experiments - RT-PCR and immunofluorescence, and carried out tail vein injections. LChen participated in the breeding, genotyping, treatment, and collection of mice. TS carried out tail vein injections and aided in lung analysis. TZ and MO created the cfms-rtTA transgenic mouse line. TB was involved in data interpretation and critically edited the manuscript. FE conceived of the study, participated in its design and coordination and helped draft the manuscript. All authors read and approved the final manuscript.
